# Synthesis, Cytotoxic Activity and 2D-QSAR Study of Some Imidazoquinazoline Derivatives

**DOI:** 10.3390/molecules19033777

**Published:** 2014-03-24

**Authors:** Hanan Georgey

**Affiliations:** Department of Pharmaceutical Chemistry, Faculty of Pharmacy, Cairo University, Cairo P.O. Box 11562, Egypt; E-Mail: hanan-hanna@hotmail.com; Tel.: +2-02-253-531-00; Fax: +2-02-253-200-05

**Keywords:** imidazolinone, imidazo[1,5-*a*]quinazoline, antitumor activity, Lipinski’s parameters, 2D-QSAR

## Abstract

A novel series of 4-substituted amino-7,8-dimethoxy-1-phenylimidazo[1,5-*a*]quinazolin-5(4*H*)-one derivatives was designed, synthesized and tested for their antitumor activity against a human mammary carcinoma cell line (MCF7). Compound **5a** was found to be the most active derivative. Physico-chemical parameters were also determined and revealed that most of the compounds obeyed the “rule of five” properties with good absorption percentages. 2D-QSAR studies revealed a well predictive and statistically significant and cross validated QSAR model that helps to explore some expectedly potent compounds.

## 1. Introduction

Struggling efforts for the treatment and eradication of cancer have grown tremendously in the last few years led to the decrease in cancer death rates by 1.8% per year in men and by 1.5% per year in women [1]. Cancer is characterized by a deregulation of the cell cycle, resulting in progressive loss of the cellular differentiation and non-controlled cellular growth [2,3]. The control of disseminated tumor growth by systemically active chemotherapeutic agents remains a major challenge for cancer chemotherapy, despite decades of focused efforts. Although there are some notable successes with certain forms of cancer [4], the discovery and development of novel therapeutic agents for the treatment of cancer has a vital importance [5,6,7,8,9,10,11].

**Figure 1:**
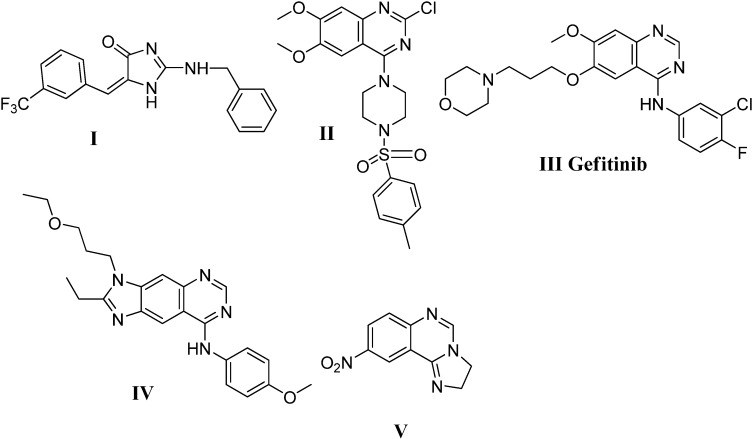
Structures of some active antitumor agents from the literature.

Imidazole, a small bioactive molecule, is a prominent structural motif found in numerous biologically active compounds. Interestingly, imidazolinones such as **I** [12] (Figure 1) and methoxyquinazoline derivatives **II**, geftinib **III** and **IV** [13,14,15,16] (Figure 1) all display significant antitumor activity. Hybrid molecules combining the imidazole and quinazoline moieties in either linear or angular imidazoquinazolines were designed and demonstrated promising antitumor activity [17,18,19]. Furthermore, incorporation of other heterocyclic moieties such as piperazine [20,21], piperidine [22,23], imidazole [24] and triazine [25] moieties was reported to enhance the antitumor activity too.

Inspired by the aforementioned facts, it was deemed of interest to use the hybridized 7,8-dimethoxyimidazoquinazoline ring system as a scaffold for the design of new molecules with potential antitumor activity. The target compounds incorporate different heterocyclic moieties such as piperazine, piperidine, or imidazole. The novel compounds were tested for their antitumor activity. Physico-chemical parameters were also calculated to obtain their “rule of five” properties. Moreover, 2D-QSAR studies were also applied to correlate between the structures of the synthesized compounds and their pharmacological activities.

## 2. Results and Discussion

### 2.1. Chemistry

**Scheme 1:**
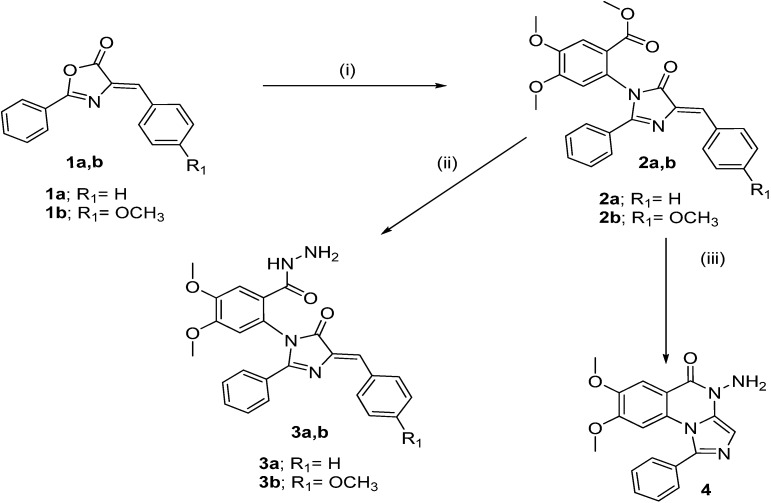
Synthesis of the target compounds **1**–**4**.

**Scheme 2:**
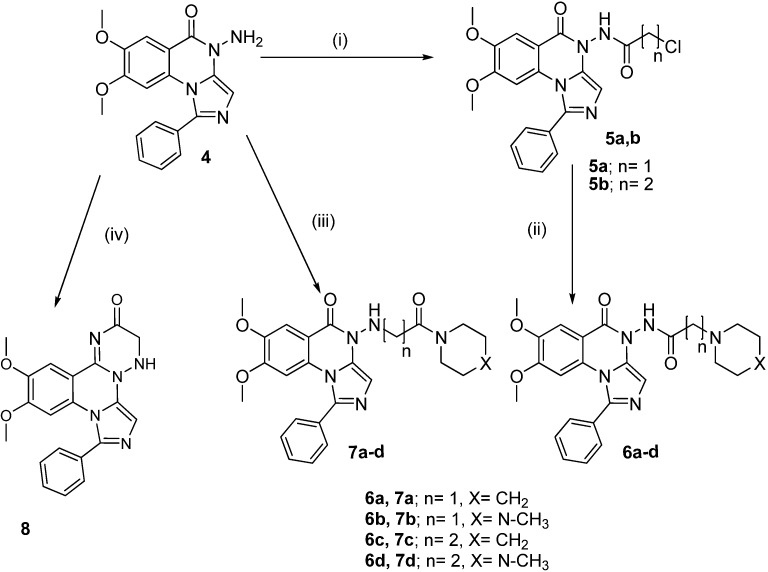
Synthesis of the target compounds **5**–**8**.

**Scheme 3:**
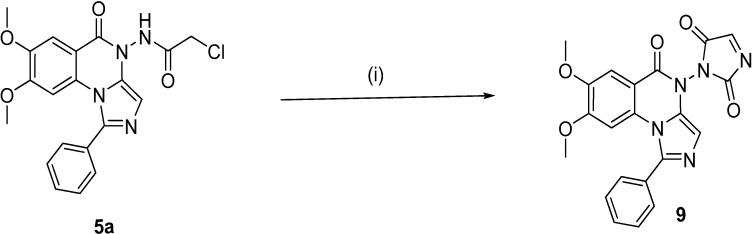
Synthesis of the target compound **9**.

The synthetic strategy for the preparation of the target compounds **2a**,**b**, **3a**,**b**, **4**, **5a**,**b**, **6a**–**d**, **7a**–**d**, **8** and **9** is illustrated in Scheme 1, Scheme 2 and Scheme 3. Imidazolinone derivatives **2a**,**b** were obtained through the reaction of oxazolone derivatives **1a**,**b**, previously synthesized according to the literature method [26,27] with methyl 2-amino-4,5-dimethoxybenzoate in glacial acetic acid in the presence of sodium acetate. This reaction proceeded through open intermediates which were recyclized in the presence of glacial acetic acid to afford the imidazolinones [28]. Reaction of **2a**,**b** with hydrazine hydrate afforded either the benzohydrazide derivatives **3a**,**b** or imidazo[1,5-*a*]quinazolinone derivative **4** depending on the reaction conditions.

*Reagents and conditions*: (i) methyl 2-amino-4,5-dimethoxybenzoate, CH_3_COOH, CH_3_COONa; (ii) Hydrazine hydrate, methanol, 2 h; (iii) Hydrazine hydrate, 80% ethanol, 24 h.

*Reagents and conditions*: (i) Cl(CH_2_)_n_COCl, dry DMF; (ii) Piperidine or N-methylpiperazine, acetonitrile, K_2_CO_3_; (iii) 2/3-chloro-1-(piperidin-1-yl/4-methylpiperazin-1-yl)ethanone/propan-1-one, dry DMF, triethylamine; (iv) 2-chloroacetamide, dry DMF.

*Reagents and conditions*: (i) KCNO, acetic acid.

Refluxing **2a**,**b** with equimolar amounts of hydrazine hydrate in methanol for 2 h yielded the benzohydrazide derivatives **3a**,**b**, whereas, the imidazoquinazoline derivative **4** was obtained according to the reported reaction conditions [29] using excess hydrazine hydrate in aqueous ethanol 80% at reflux for 24 h. (Scheme 1). On the other hand, the reaction of compound **4** with chloroacetyl chloride in DMF led to the formation of 2-chloro-*N*-(7,8-dimethoxy-5-oxo-1-phenylimidazo[1,5-*a*]quinazolin-5(4*H*)-yl)acetamide (**5a**). Similarly, the reaction with chloropropionyl chloride gave **5b**. Reaction of **5a**,**b** with secondary amines (namely, piperidine and *N*-methylpiperazine) in dry acetonitrile in the presence of potassium carbonate yielded compounds **6a**–**d**. Ethylamino or propylamino-1-phenylimidazo[1,5-*a*]quinazolin-5(4*H*)-one derivatives **7a**–**d** were obtained by reacting compound **4** with 2/3-chloro-1-(piperidin-1-yl/4-methylpiperazin-1-yl)ethanone/propan-1-one in dry DMF in the presence of triethylamine. Moreover, formation of the oxotriazine ring was achieved via the reaction of the *N*-amino derivative **4** with chloroacetamide in DMF to yield 11,12-dimethoxy-8-phenyl-3,4-dihydro-2*H*-imidazo[1,5-*a*][1,2,4]triazino[2,3-c]quinazolin-2-one (**8**) as depicted in Scheme 2. As shown in Scheme 3, the cyclized product **9** was obtained starting with the chloroacetamide derivative **5a**. Reaction with potassium cyanate yielded the 1-(7,8-dimethoxy-5-oxo-1-phenylimidazo[1,5-*a*]quinazolin-4(5*H*)-yl)-1*H*-imidazole-2,5-dione(**9**). The newly synthesized compounds were confirmed via elemental analyses and spectral data (IR, ^1^H-NMR, ^13^C-NMR and mass spectra).

### 2.2. Preliminary in-Vitro Antitumor Screening

All the synthesized compounds were tested for their antitumor activity against human mammary carcinoma cell line (MCF7) at the National Cancer Institute, Cairo University [30]. From the observed antitumor data (Table 1), it was noticed that imidazolinone derivative **3a** and imidazoquinazoline derivatives **5a**, **6a**, **7a** and **7b** showed IC_50_ values of 27.1, 10.6, 26.4, 21.6 and 26.8 µM, respectively. Also, compounds **5a** and **7a** revealed remarkable activity comparable to that of the reference drug mitoxantrone.

Structure-activity correlations, based on the human mammary carcinoma cell line (MCF7), revealed that the chloroacetyl derivative **5a** was more active than its chloropropionyl analogue **5b**. Regarding the effect of the substituent on **5a**,**b**, the results of **6a**–**d** and **7a**–**d** showed that a piperidinyl moiety was more active than a *N*-methylpiperazinyl one, and an acetyl moiety was more active than its corresponding propionyl moiety. Also, derivatives in which the carbonyl moiety was directly attached to a piperidinyl/piperazinyl (compounds **7a**–**d**) were more active than those in which the carbonyl was attached to an *N*-aminoimidazoquinazoline (compounds **6a**–**d**).

**Table 1:** Cytotoxic activity of the newly synthesized compounds against breast cancer MCF7 cell line.

Compd. No	IC_50_ µg/mL	IC_50_ µM
**2a**	18.0	40.6
**2b**	21.8	46.1
**3a**	12.0	27.1
**3b**	42.4	89.7
**4**	12.2	36.2
**5a**	4.4	10.6
**5b**	14.0	32.8
**6a**	12.2	26.4
**6b**	17.2	36.0
**6c**	19.0	39.9
**6d**	21.4	43.6
**7a**	10.0	21.6
**7b**	12.8	26.8
**7c**	17.4	36.5
**7d**	18.4	37.5
**8**	21	56.1
**9**	19.0	45.5
**Mitoxantrone**	10.1	22.7

### 2.3. Physico-Chemical Parameters

Computational study for the prediction of physico-chemical parameters of all synthesized compounds has been executed. The computed molecular properties are shown in Table 2. Numbers of rotatable bonds, log *P*, molecular polar surface area (PSA), number of hydrogen bond accepter and donor atoms of Lipinski’s “rule of five” [31] were calculated using the Molinspiration online property calculation toolkit [32]. The degree of absorption is expressed by the percentage of absorption (%ABS), that was calculated by using %ABS = 109 − (0.345 × TPSA) [33]. From all these parameters, it was found that, all the synthesized derivatives exhibited %ABS ranging from 61.41% to 84.91%. Furthermore, most of the compounds obeyed the “rule of five” properties.

### 2.4. 2D-QSAR Studies

A 2D-QSAR study was performed in order to find a mathematical correlation between the structures of the newly synthesized compounds and their antitumor activity [34] that expressed as –log IC_50_ values, using Molecular Operating Environment (MOE) software package.

**Table 2:** Calculated absorption (%ABS), polar surface area (PSA) and Lipinski parameters of the newly synthesized compounds.

Compd. No	%ABS	TPSA	nrotb	nON ≤ 10	nOHNH ≤ 5	miLog *P* ≤ 5	MW ≤ 500	n violations ≤ 1
**2a**	81.51	79.67	7	7	0	4.38	444.15	0
**2b**	78.32	88.90	8	8	0	4.43	472.16	0
**3a**	71.57	108.48	6	8	3	2.93	442.16	0
**3b**	68.38	117.72	7	9	3	2.98	472.49	0
**4**	79.08	83.80	3	7	2	2.34	336.35	0
**5a**	79.02	86.87	5	8	1	2.64	412.82	0
**5b**	79.02	86.87	6	8	1	2.91	426.10	0
**6a**	77.91	90.11	6	9	1	2.97	461.51	0
**6b**	76.79	93.35	6	10	1	1.96	476.52	0
**6c**	77.91	90.11	7	9	1	3.24	475.53	0
**6d**	76.79	93.35	7	10	1	2.23	490.55	0
**7a**	77.91	90.11	6	9	1	2.97	461.51	0
**7b**	76.79	93.35	6	10	1	1.96	476.52	0
**7c**	77.91	90.11	7	9	1	3.24	475.53	0
**7d**	76.79	93.35	7	10	1	2.23	490.55	0
**8**	80.65	82.17	3	8	1	2.61	375.38	0
**9**	71.13	109.74	4	10	0	1.67	417.37	0

The most relevant descriptors derived for modeling the antitumor activity are listed in Table 3 and the relation between the obtained results of experimentally observed and predicted values of antitumor activity were presented in Table 4. 2D-QSAR models were validated with the leave one out (LOO) method. The best derived QSAR model for the newly synthesized derivatives was presented by the following triparametric equation with correlation coefficient (R^2^) = 0.92:

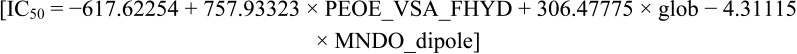
(1)
where *n* = 9, RMSE = 3.4641, *R^2^* = 0.92722, *n*: number of compounds used for construction of model; RMSE: Root mean square error; *R^2^*: correlation coefficient.

From this model, it was observed that PEOE_VSA_FHYD (Fractional hydrophobic Van der Waal surface area) and glob (Globularity, or inverse condition number (smallest eigenvalue divided by the largest eigen value) of the covariance matrix of atomic coordinates. A value of 1 indicates a perfect sphere while a value of 0 indicates a two- or one-dimensional object) were positively correlated with antitumor activity but MNDO_dipole (The dipole moment calculated using the MNDO Hamiltonian [MOPAC]) was negatively correlated with activity indicated that increasing in the values PEOE_VSA_FHYD is beneficial for antitumor activity and the experimental IC_50_ was matched with the expected IC_50_.

**Table 3:** The most relevant discriptors derived for modeling the antitumor activity.

Compd. No	*P* IC_50_	PEOE_VS	Glob	MNDO_di
**4**	36.2	0.8547	0.0796	3.7779
**5a**	10.6	0.8258	0.0670	4.2328
**5b**	32.8	0.8332	0.0725	4.3450
**6a**	26.4	0.8517	0.0514	1.6865
**6b**	36.0	0.8602	0.0519	0.0000
**6c**	39.9	0.8571	0.1028	5.6151
**6d**	43.6	0.8650	0.0387	1.9895
**7a**	21.6	0.8517	0.0389	5.1133
**7b**	26.8	0.8602	0.0591	5.0499
**7c**	36.5	0.8517	0.0690	3.9824

**Table 4:** Experimentally observed, predicted activity of the new compounds and their residual.

Compd. No	Activity *P* IC_50_	$PRED	$RES
**4**	36.2	38.2548	−2.0548
**5a**	10.6	10.5372	0.0628
**5b**	32.8		
**6a**	26.4	36.3854	−9.9854
**6b**	36.0	50.2617	−14.2617
**6c**	39.9	39.2977	0.6023
**6d**	43.6	41.2839	2.3161
**7a**	21.6	17.7661	3.8339
**7b**	26.8	30.6762	−3.8762
**7c**	36.5	35.9551	0.5448

## 3. Experimental

### 3.1. General Information

Unless specified all chemicals were of commercial grade, obtained from Aldrich Chemical Co. (Milwaukee, WI, USA), and used without further purification. Melting points were carried out by the open capillary tube method using a Gallenkamp digital melting point Griffin apparatus 1901 and they were uncorrected. Elemental microanalyses were recorded at the Regional Center of Mycology and Biotechnology, Al-Azhar University. Infrared Spectra were recorded on Shimadzu infrared spectrophotometer IR Affinity-1 (FTIR-8400S, Kyoto, Japan) and expressed in wave number (cm^−1^), using potassium bromide discs. NMR spectra were recorded on a Varian Mercury VX 300 spectrometer (^1^H: 300, ^13^C APT: 75 MHz) or a 400 MHz high performance digital FT-NMR spectrophotometer using TMS as an internal standard. The exchangeable protons (NH, and OH) were exchanged by D_2_O. Mass Spectra was recorded on Shimadzu QP-2010 plus. All reactions were monitored by thin layer chromatography. Silica gel/TLC-cards DC-Alufolien-Kieselgel with fluorescent indicator 254 nm; layer thickness 0.2 mm; 20 × 20 cm aluminum cards were used. Petroleum ether/ethyl acetate (1:1) or (1:2) was the adopted solvent system. Compounds **1a**,**b** [26,27] were prepared according to reported procedures.

### 3.2. Chemistry

#### 3.2.1. General Procedure for the Preparation of **2a**, **b**

A mixture of **1a**, **b** (10 mmol) and methyl 2-amino-4,5-dimethoxybenzoate (12 mmol, 2.52 g), freshly prepared fused sodium acetate (0.3 g) was heated in a boiling water bath in glacial acetic acid (10 mL) for 3 h. The crystalline product separated on cooling was filtered, washed with water, dried, and recrystallized from ethanol.

*Methyl 2-(4-benzylidene-5-oxo-2-phenyl-4,5-dihydroimidazol-1-yl)-4,5-dimethoxybenzoate* (**2a**). Yield: 81%; mp 221–223 °C; IR (KBr, cm^−1^): 3086 (CH aromatic), 2958, 2935 (CH aliphatic), 1693, 1674 (C=O); ^1^H-NMR (DMSO-*d*_6_): *δ* 3.65 (s, 3H, COOCH_3_), 3.77 (s, 3H, OCH_3_), 3.88 (s, 3H, OCH_3_), 7.36–7.43 (m, 4H, benzyl. 3,4,5-CHs + benzo.6-CH) 7.54–7.63 (m, 6H, phenyl CHs + benzo.3-CH), 8.04 (d, 2H, *J* = 6.9, benzyl. 2,6-CHs), 8.57 (s, 1H, CH); ^13^ C-NMR (DMSO): 52.4, 56.09, 56.1, 103.4, 107.1, 112.6, 128.4, 128.7, 129.1, 129.7, 130.0, 130.4, 131.9, 132.3, 134.0, 137.1, 144.2, 154.0, 163.9, 167.4, 167.8; MS, *m/z* (%): M^+^, 442 (23.54%), 105 (100%). Anal. Calcd. for C_26_H_22_N_2_O_5_ (442.46): C, 70.58; H, 5.01; N, 6.33. Found: C, 70.32; H, 5.12; N, 6.44%.

*Methyl 4,5-dimethoxy-2-(4-(4-methoxybenzylidene)-5-oxo-2-phenyl-4,5-dihydro-1H-imidazol-1-yl)- benzoate* (**2b**). Yield: 84%; mp 211–212 °C; IR (KBr, cm^−1^): 3062 (CH aromatic), 2939, 2839 (CH aliphatic), 1720, 1678 (C=O); ^1^H-NMR (DMSO-*d*_6_): *δ* 3.63 (s, 3H, COOCH_3_), 3.84 (s, 6H, 2OCH_3_), 3.86 (s, 3H, OCH_3_), 6.95 (d, 2H, *J* = 9, benzyl. 3, 5-CHs), 7.39 (s, 1H, benzo.6-CH) 7.54–7.63 (m, 6H, phenyl CHs + benzo.3-CH), 8.04 (d, 2H, *J* = 6.9, benzyl. 2,6-CHs), 8.58 (s, 1H, CH); ^13^C-NMR (DMSO): 51.8, 55.5, 55.7, 56.1, 102.7, 106.4, 112.5, 114.4, 126.0, 127.6, 128.2, 130.5, 131.4, 131.7, 133.5, 134.2, 136.7, 143.5, 153.4, 160.0, 163.5, 166.8, 167.2; MS, *m/z* (%): M^+^, 472 (68.44%), 105 (100%). Anal. Calcd. for C_27_H_24_N_2_O_6_ (472.48): C, 68.63; H, 5.12; N, 5.93. Found: C, 68.85; H, 5.00; N, 6.01%.

#### 3.2.2. General Procedure for the Preparation of **3a**, **b**

A solution of **2a**, **b** (10 mmol), hydrazine hydrate (12 mmol, 0.60 g) in methanol (50 mL) was refluxed for 2 h. The mixture was concentrated under vacuum, and water was added to the residue, which was filtered, dried, and crystallized from ethanol.

*2-(4-Benzylidene-5-oxo-2-phenyl-4,5-dihydroimidazol-1-yl)-4,5-dimethoxybenzo- hydrazide* (**3a**). Yield: 71%; mp 151–153 °C; IR (KBr, cm^−1^): 3456, 3356, 3305 (NH_2_, NH), 3059 (CH aromatic), 2997, 2951 (CH aliphatic), 1674 (C=Os); ^1^H-NMR (DMSO-*d*_6_): *δ* 3.73 (s, 3H, OCH_3_), 3.79 (s, 3H, OCH_3_), 6.45 (s, 2H, NH_2_), 7.11–7.38 (m, 4H, benzyl. 3,4,5-CHs + benzo.6-CH), 7.42–7.49 (m, 6H, phenyl CHs + benzo.3-CH), 7.79 (d, 2H, *J* = 6.9, benzyl. 2,6-CHs), 8.59 (s, 1H, CH), 9.33 (s, 1H, NH); ^13^C-NMR (DMSO): 55.1, 55.9, 99.0, 112.2, 126.1, 127.3, 127.9, 128.0, 129.0, 131.1, 133.9, 138.2, 139.2, 143.5, 148.2, 154.7, 166.0, 167.2, 170.5; MS, *m/z* (%): M^+^, 442 (0.25%), 50 (100%). Anal. Calcd. for C_25_H_22_N_4_O_4_ (442.46): C, 67.86; H, 5.01; N, 12.66. Found: C, 67.86; H, 5.12; N, 12.93%.

*2-(4-(4-Methoxybenzylidene)-5-oxo-2-phenyl-4,5-dihydroimidazol-1-yl)-4,5-dimethoxybenzohydrazide* (**3b**). Yield: 74%; mp 201–203 °C; IR (KBr, cm^−1^): 3441, 3244, 3200 (NH_2_, NH), 3050 (CH aromatic), 2924, 2835 (CH aliphatic), 1674, 1658 (C=Os); ^1^H-NMR (DMSO-*d*_6_): *δ* 3.71 (s, 3H, OCH_3_), 3.80 (s, 3H, OCH_3_), 3.86 (s, 3H, OCH_3_), 6.88 (s, 2H, NH_2_), 6.98 (d, 2H, *J* = 8.7, benzyl. 3, 5-CHs), 7.39 (s, 1H, benzo.6-CH), 7.55–7.63 (m, 6H, phenyl CHs + benzo.3-CH), 8.04 (d, 2H, *J* = 6.9, benzyl. 2,6-CHs), 8.60 (s, 1H, CH), 10.30 (s, 1H, NH); ^13^C-NMR (DMSO): 51.8, 55.1, 55.3, 102.5, 112.8, 125.7, 126.4, 127.7, 128.3, 130.0, 131.4, 132.6, 133.1, 136.0, 143.4, 153.1, 154.7, 160.05, 163.5, 167.2, 167.5; MS, *m/z* (%): M^+^, 472 (0.38%), 57 (100%). Anal. Calcd. for C_26_H_24_N_4_O_5_ (472.49): C, 66.09; H, 5.12; N, 11.86. Found: C, 66.34; H, 5.45; N, 12.24%.

#### 3.2.3. Synthesis of 4-Amino-7,8-dimethoxy-1-phenylimidazo[1,5-*a*]quinazolin-5(4*H*)-one (**4**)

To a suspension of the appropriate **2a, b** (10 mmol) in aqueous ethanol (80%), hydrazine hydrate (30 mmol, 1.50 g) was added. The mixture was refluxed for 24 h. The resulting solution was distilled under vacuum and the residue was triturated with ice water. The separated solid was filtered, washed with water, and crystallized from ethanol. Yield: 64% from **2a**, 72% from **2b**; mp 281–283 °C; IR (KBr, cm^−1^): 3317, 3267 (NH_2_), 3074 (CH aromatic), 2927, 2839 (CH aliphatic), 1680 (C=O); ^1^H-NMR (DMSO-*d*_6_): *δ* 3.89 (s, 3H, OCH_3_), 3.90 (s, 3H, OCH_3_), 4.42 (s, 2H, NH_2_), 7.19 (s, 1H, imidazo.9-CH), 7.45–7.55 (m, 7H, Ar-H); ^13^C-NMR (DMSO): 55.7, 55.9, 105.0, 107.9, 113.1, 127.8, 128.0, 128.3, 129.5, 135.1, 143.9, 148.7, 154.5, 154.7, 160.3, 166.3; MS, *m/z* (%): M^+^+1, 337 (0.25%), 196 (100%). Anal. Calcd. for C_18_H_16_N_4_O_3_ (336.34): C, 64.28; H, 4.79; N, 16.66. Found: C, 64.37; H, 4.86; N, 16.92%.

#### 3.2.4. General Procedure for the Preparation of **5a**, **b**

A solution of compound **4** (5 mmol, 1.68 g) in dry DMF (5 mL) containing chloroacetylchloride or chloropropionyl chloride (5.5 mmol) was stirred at room temperature (25–30 °C) for 24 h. The solution was poured onto crushed ice and the resulting solid was filtered, washed and crystallized from ethanol.

*2-Chloro-N-(7,8-dimethoxy-5-oxo-1-phenylimidazo[1,5-a]quinazolin-5(4H)-yl) acetamide* (**5a**). Yield: 82%; mp 176–178 °C; IR (KBr, cm^−1^): 3224 (NH), 3035 (CH aromatic), 2947, 2854 (CH aliphatic), 1674 (C=O); ^1^H-NMR (DMSO-*d*_6_): *δ* 3.81 (s, 3H, OCH_3_), 3.86 (s, 3H, OCH_3_), 4.44 (s, 2H, CH_2_), 7.40–8.20 (m, 8H, Ar-H), 11.47 (s, 1H, NH); ^13^C-NMR (DMSO): 43.3, 55.4, 55.7, 103.3, 107.6, 112.0, 127.3, 128.0, 128.2, 129.0, 135.0, 144.1, 144.2, 153.1, 153.4, 164.9, 167.0; MS, *m/z* (%): M^+^+2, 414 (0.31%), 412 (0.91%), 105 (100%). Anal. Calcd. for C_20_H_17_ClN_4_O_4_ (412.82): C, 58.19; H, 4.15; N, 13.57. Found: C, 58.34; H, 4.01; N, 13.78%.

*3-Chloro-N-(7,8-dimethoxy-5-oxo-1-phenylimidazo[1,5-a]quinazolin-5(4H)-yl) propamide* (**5b**). Yield: 75%; mp 209–210 °C; IR (KBr, cm^−1^): 3248 (NH), 3055 (CH aromatic), 2951, 2824 (CH aliphatic), 1670 (C=O); ^1^H-NMR (DMSO-*d*_6_): *δ* 2.88 (t, 2H, CH_2_, *J* = 7.8), 3.79 (t, 2H, CH_2_, *J* = 7.8), 3.90 (s, 3H, OCH_3_), 3.92 (s, 3H, OCH_3_), 7.15–8.60 (m, 8H, Ar-H), 10.7 (s, 1H, NH); ^13^C-NMR (DMSO): 36.0, 52.2, 54.4, 55.7, 103.9, 108.2, 112.9, 127.1, 128.0, 128.2, 128.9,131.5, 133.3, 135.0, 137.5, 143.8, 152.8, 166.7, 170.4; MS, *m/z* (%): M^+^, 426 (0.22%), 104 (100%). Anal. Calcd. for C_21_H_19_ClN_4_O_4_ (426.85): C, 59.09; H, 4.49; N, 13.13. Found: C, 59.18; H, 4.53; N, 13.47%.

#### 3.2.5. General Procedure for the Preparation of **6a**–**d**

A mixture of equimolar amounts of the appropriate **5a**,**b** and the corresponding secondary amine (2 mmol) in dry acetonitrile (30 mL) containing potassium carbonate (4 mmol, 0.55 g) was refluxed for 12 h and the reaction mixture was filtered hot. The solid which was separated upon storing the clear reaction mixture at room temperature overnight was collected and crystallized from ethanol.

*N-(7,8-Dimethoxy-5-oxo-1-phenylimidazo[1,5-a]quinazolin-5(4H)-yl)-2-(piperidin-1-yl)acetamide* (**6a**). Yield: 77%; mp 186–189 °C; IR (KBr, cm^−1^): 3217 (NH), 3016 (CH aromatic), 2943, 2879 (CH aliphatic), 1693, 1670 (C=Os); ^1^H-NMR (DMSO-*d*_6_): *δ* 1.42–1.43 (m, 2H, CH_2_), 1.63–1.68 (m, 4H, 2CH_2_), 2.46 (t, 4H, 2CH_2_, *J* = 8.7), 3.07 (s, 2H, CH_2_), 3.80 (s, 3H, OCH_3_), 3.86 (s, 3H, OCH_3_), 7.40–8.40 (m, 8H, Ar-H), 11.8 (s, 1H, NH); ^13^C-NMR (DMSO): 23.4, 25.2, 52.2, 55.4, 55.4, 62.9, 102.9, 106.8, 112.1, 127.4, 128.1, 128.3, 129.0, 135.9, 144.3, 144.4, 153.1, 153.4, 166.5, 169.6; MS, *m/z* (%): M^+^, 461 (47.5%), 254 (100%). Anal. Calcd. for C_25_H_27_N_5_O_4_ (461.51): C, 65.06; H, 5.90; N, 15.17. Found: C, 65.38; H, 5.93; N, 15.42%.

*N-(7,8-Dimethoxy-5-oxo-1-phenylimidazo[1,5-a]quinazolin-5(4H)-yl)-2-(4-methylpiperazin-1-yl)acetamide* (**6b**). Yield: 81%; mp 167–168 °C; IR (KBr, cm^−1^): 3205 (NH), 3030 (CH aromatic), 2985, 2947, 2816 (CH aliphatic), 1689, 1670 (C=Os); ^1^H-NMR (DMSO-*d*_6_): *δ* 2.19 (s, 3H, CH_3_), 2.46–2.53 (m, 8H, 4CH_2_), 3.75 (s, 2H, CH_2_), 3.80 (s, 3H, OCH_3_), 3.86 (s, 3H, OCH_3_), 7.18–8.41 (m, 8H, Ar-H), 11.76 (s, 1H, NH); ^13^C-NMR (DMSO): 46.2, 52.5, 53.3, 54.7, 54.9, 56.0, 56.1, 62.5, 103.6, 107.5, 112.7, 127.9, 128.6, 129.05, 136.4, 144.0, 153.7, 167.0, 169.8; MS, *m/z* (%): M^+^, 476 (19.9%), 55 (100%). Anal. Calcd. for C_25_H_28_N_6_O_4_ (476.52): C, 63.01; H, 5.92; N, 17.64. Found: C, 63.13; H, 6.12; N, 17.79%.

*N-(7,8-Dimethoxy-5-oxo-1-phenylimidazo[1,5-a]quinazolin-5(4H)-yl)-3-(piperi-din-1-yl)propamide* (**6c**). Yield: 66%; mp 105–108 °C; IR (KBr, cm^−1^): 3263 (NH), 3010 (CH aromatic), 2931, 2894 (CH aliphatic), 1674 (br., C=O); ^1^H-NMR (DMSO-*d*_6_): *δ* 1.37–1.48 (m, 6H, 3CH_2_), 2.53–2.55 ( m, 4H, 2CH_2_), 2.61 (t, 2H, 2CH_2_, *J* = 6.3), 3.69 (t, 2H, 2CH_2_, *J* = 9.3), 3.90 (s, 3H, OCH_3_), 3.91 (s, 3H, OCH_3_), 7.20–8.10 (m, 8H, Ar-H), 10.85 (s, 1H, NH); ^13^C-NMR (DMSO): 22.2, 25.6, 35.2, 52.6, 53.7, 54.4, 55.9, 56.0, 56.1, 104.6, 108.4, 112.6, 127.4, 127.9, 128.2, 128.6, 128.7, 129.1, 136.3, 144.2, 153.6, 167.6, 170.7; MS, *m/z* (%): M^+^, 475 (0.5%), 98 (100%). Anal. Calcd. for C_26_H_29_N_5_O_4_ (475.53): C, 65.67; H, 6.15; N, 14.73. Found: C, 65.46; H, 5.84; N, 14.50%.

*N-(7,8-Dimethoxy-5-oxo-1-phenylimidazo[1,5-a]quinazolin-5(4H)-yl)-3-(4-methylpiperazin-1-yl)-propamide* (**6d**). Yield: 69%; mp 130–132 °C; IR (KBr, cm^−1^): 3217 (NH), 3059 (CH aromatic), 2939, 2835 (CH aliphatic), 1674 (br., C=O); ^1^H-NMR (DMSO-*d*_6_): *δ* 2.12 (s, 3H, CH_3_), 2.28–2.45 (m, 8H, 4CH_2_), 2.62 (t, 2H, 2CH_2_, *J* = 6.3), 3.72 (t, 2H, CH_2_, *J* = 7.8), 3.90 (s, 3H, OCH_3_), 3.92 (s, 3H, OCH_3_), 7.19–8.24 (m, 8H, Ar-H), 10.84 (s, 1H, NH); ^13^C-NMR (DMSO): 34.4, 44.5, 51.1, 52.8, 54.3, 55.3, 103.9, 106.2, 111.7, 127.1, 128.3, 128.8, 129.5, 135.1, 143.5, 144.4, 152.7, 153.4, 167.0, 170.4; MS, *m/z* (%): M^+^, 490 (0.11%), 70 (100%). Anal. Calcd. for C_26_H_30_N_6_O_4_ (490.55): C, 63.66; H, 6.16; N, 17.13. Found: C, 64.13; H, 6.28; N, 17.46%.

#### 3.2.6. General Procedure for the Preparation of **7a**–**d**

A mixture of equimolar amounts of compound **4** and the corresponding 2/3-chloro-1-(piperidin-1-yl/4-methylpiperazin-1-yl) ethanone/propan-1-one (2 mmol) in dry DMF (10 mL) containing triethylamine (0.5 mL) was refluxed for 10 h. The resulting solution was cooled and poured onto crushed ice. The separated solid was filtered, dried, and crystallized from methanol.

*7,8-Dimethoxy-4-(2-oxo-2-piperidin-1-yl)ethylamino)-1-phenylimidazo[1,5-a] quinazolin-5(4H)-one* (**7a**). Yield: 77%; mp 142–143 °C; IR (KBr, cm^−1^): 3232 (NH), 3056 (CH aromatic), 2923, 2854 (CH aliphatic), 1670, 1654 (C=O); ^1^H-NMR (DMSO-*d*_6_): *δ* 1.27–1.38 (m, 2H, CH_2_), 1.42–1.56 (m, 4H, 2CH_2_), 3.11–3.28 (m, 4H, 2CH_2_), 3.73 (s, 2H, CH_2_), 3.89 (s, 3H, OCH_3_), 3.91 (s, 3H, OCH_3_), 6.45 (s, 1H, NH), 7.17–8.87 (m, 8H, Ar-H); ^13^C-NMR (DMSO): 21.3, 21.8, 45.2, 51.0, 55.1, 55.7, 98.9, 99.6, 112.0, 127.2, 128.1, 128.2, 128.8, 131.5, 139.2, 147.7, 153.4, 154.6, 167.4; MS, *m/z* (%): M^+^, 461 (0.51%), 196 (100%). Anal. Calcd. for C_25_H_27_N_5_O_4_ (461.51): C, 65.06; H, 5.90; N, 15.17. Found: C, 65.11; H, 5.97; N, 15.24%.

*7,8-Dimethoxy-4-(2-(4-methylpiperazin-1-yl)-2-oxo-ethylamino)-1-phenylimidazo[1,5-a]quinazolin-5(4H)-one* (**7b**). Yield: 67%; mp 160–161 °C; IR (KBr, cm^−1^): 3305 (NH), 3059 (CH aromatic), 2924, 2854 (CH aliphatic), 1670, 1661 (C=O); ^1^H-NMR (DMSO-*d*_6_): *δ* 2.22–2.34 (m, 7H, CH_3_ and 2CH_2_), 3.16 (t, 4H, 2CH_2_, *J* = 6.3), 3.507 (s, 2H, CH_2_), 3.89 (s, 3H, OCH_3_), 3.90 (s, 3H, OCH_3_), 6.44 (s, 1H, NH), 7.09–8.09 (m, 8H, Ar-H); ^13^C-NMR (DMSO): 44.5, 51.2, 52.1, 53.5, 54.3, 55.3, 103.9, 106.4, 111.8, 127.2, 128.3, 128.8, 129.3, 130.1, 135.2, 143.6, 144.7, 152.7, 153.4, 167.0, 170.4; MS, *m/z* (%): M^+^, 476 (0.22%), 105 (100%). Anal. Calcd. for C_25_H_28_N_6_O_4_ (476.52): C, 63.01; H, 5.92; N, 17.64. Found: C, 62.98; H, 6.23; N, 17.81%.

*7,8-Dimethoxy-4-(3-oxo-2-piperidin-1-yl)propylamino)-1-phenylimidazo[1,5-a]quinazolin-5(4H)-one* (**7c**). Yield: 69%; mp 98–100 °C; IR (KBr, cm^−1^): 3255 (NH), 3028 (CH aromatic), 2927, 2854 (CH aliphatic), 1672, 1654 (C=O); ^1^H-NMR (DMSO-*d*_6_): *δ* 1.19–1.27 (m, 2H, CH_2_), 1.44–1.56 (m, 4H, 2CH_2_), 2.72 (t, 2H, CH_2_, *J* = 11.1), 2.97 (t, 2H, 2CH_2_, *J* = 11.4), 3.43–3.69 (t, 4H, 2CH_2_, *J* = 6.6), 3.81 (s, 3H, OCH_3_), 3.91 (s, 3H, OCH_3_), 6.46 (s, 1H, NH),7.13–8.08 (m, 8H, Ar-H); ^13^C-NMR (DMSO): 23.8, 24.6, 25.9, 44.5, 51.0, 55.1, 55.7, 98.9, 99.6, 112.0, 127.1, 128.1, 128.2, 129.0, 133.2, 139.2, 147.7, 153.4, 154.6, 162.8, 167.4; MS, *m/z* (%): M^+^, 475 (0.52%), 207 (100%). Anal. Calcd. for C_26_H_29_N_5_O_4_ (475.53): C, 65.67; H, 6.15; N, 14.73. Found: C, 65.78; H, 6.21; N, 14.92%.

*7,8-Dimethoxy-4-(3-(4-methylpiperazin-1-yl)-3-oxo-propylamino)-1-phenylimidazo[1,5-a]quinazolin-5(4H)-one* (**7d**). Yield: 69% mp 210–211 °C; IR (KBr, cm^−1^): 3217 (NH), 3056 (CH aromatic), 2924, 2850 (CH aliphatic), 1674, 1658 (C=O); ^1^H-NMR (DMSO-*d*_6_): *δ* 2.12–2.43 (m, 7H, CH_3_ and 2CH_2_), 2.72 (t, 2H, CH_2_, *J* = 11.1), 2.88 (t, 2H, CH_2_, *J* = 11.4), 3.05 (t, 4H, 2CH_2_, *J* = 7.2), 3.89 (s, 3H, OCH_3_), 3.90 (s, 3H, OCH_3_), 6.45 (s, 1H, NH)7.19–8.14 (m, 8H, Ar-H),; ^13^C-NMR (DMSO): 31.1, 36.1, 45.8, 51.4, 55.6, 56.4, 99.5, 100.1, 112.8, 127.8, 127.9, 128.6, 128.9, 129.3, 139.8, 148.7, 155.3, 162.8, 167.8; MS, *m/z* (%): M^+^, 490 (22.1%), 70 (100%). Anal. Calcd. for C_26_H_30_N_6_O_4_ (490.55): C, 63.66; H, 6.16; N, 17.13. Found: C, 63.81; H, 6.30; N, 17.36%.

#### 3.2.7. Synthesis of 11,12-Dimethoxy-8-phenyl-3,4-dihydro-2*H*-imidazo[1,5-*a*][1,2,4]triazino[2,3-c]quinazolin-2-one (**8**)

A mixture of **4** (10 mmol, 3.36 g) and 2-chloracetamide (12 mmol, 1.12 g) in dry DMF (15 mL) was refluxed for 24 h. The desired product was obtained through pouring the reaction solution onto ice water and the precipitate was collected, washed, dried, and then crystallized from DMF/water Yield: 61%; mp 190–192 °C; IR (KBr, cm^−1^): 3210 (NH), 3011 (CH aromatic), 2958, 2924 (CH aliphatic), 1666 (C=O); ^1^H-NMR (DMSO-*d*_6_): *δ* 3.74 (s, 2H, CH_2_), 3.89 (s, 3H, OCH_3_), 3.93 (s, 3H, OCH_3_), 5.04 (s, 1H, NH),7.29–8.31 (m, 8H, Ar-H); ^13^C-NMR (DMSO): 51.5, 55.6, 56.4, 101.4, 112.8, 126.1, 127.9, 128.1, 128.6, 129.2, 129.5, 131.9, 134.2, 138.4, 146.8, 155.3, 168.0; MS, *m/z* (%): M^+^−1, 374 (8.6%), 50 (100%). Anal. Calcd. for C_20_H_17_N_5_O_3_ (375.38): C, 63.99; H, 4.56; N, 18.66. Found: C, 63.81; H, 4.78; N, 18.24%.

#### 3.2.8. Synthesis of 1-(7,8-Dimethoxy-5-oxo-1-phenylimidazo[1,5-*a*]quinazolin-4(5*H*)-yl)-1*H*-imidazole-2,5-dione (**9**)

A suspension of **5a** (5 mmol, 2.06 g) and potassium cyanate (10 mmol, 0.81 g) in acetic acid (10 mL) was refluxed for 12 h. The mixture was concentrated under vacuum; water was added to the residue, filtered, dried, and crystallized from ethanol. Yield: 82%; mp 202–203 °C; IR (KBr, cm^−1^): 3030 (CH aromatic), 2985, 2927 (CH aliphatic), 1670 (C=O); ^1^H-NMR (DMSO-*d*_6_): *δ* 3.90 (s, 3H, OCH_3_), 3.92 (s, 3H, OCH_3_), 7.50–8.35 (m, 9H, Ar-H); ^13^C-NMR (DMSO): 52.3, 55.5, 103.5, 107.8,112.0, 127.9, 128.1, 128.3, 129.5, 135.0, 144.1, 148.7, 153.1, 154.7, 164.9, 167.0, 167.1; MS, *m/z* (%): M^+^, 417 (3.05%), 55 (100%). Anal. Calcd. for C_21_H_15_N_5_O_5_ (417.37): C, 60.43; H, 3.62; N, 16.78. Found: C, 60.21; H, 3.71; N, 16.47%.

### 3.3. In Vitro Antitumor Activity Measurement against Human Mammary Carcinoma Cell Line (MCF7)

Cells were plated in 96-multiwell plate (104 cells/well) for 24 h before treatment with the compounds to allow attachment of cell to the wall of the plate. Different concentrations of the compound under test (0, 1, 2.5, 5 and 10 µg/mL) were added to the cell monolayer. Triplicate wells were prepared for each individual dose. Monolayer cells were then incubated with the compounds for 48 h at 37 °C and in atmosphere of 5% CO_2_. After this time, cells were fixed, washed, and stained with Sulforhodamine B stain. Excess stain was washed with acetic acid and attached stain was recovered with Tris EDTA buffer. The color intensity was measured in an ELISA reader. Finally, the relation between surviving fraction and drug concentration was plotted to get the survival curve of the tumor cell line after the specific compound [30]. The optical density measured is linear to the cell number of the surviving fraction. Therefore, the assay is a sensitive measure of compound induced cytotoxicity with the best signal to noise ratio. The assay also, provides a colorimeteric end point that is nondestructive, indefinitely stable and visible to naked eye.

### 3.4. 2D-QSAR Study

#### 3.4.1. Data Set

All the molecular modeling calculations and docking simulation studies were performed using Molecular Operating Environment (MOE^®^) version 10.2010, Chemical Computing Group Inc., Montreal, Canada. The computational software operated under Windows XP installed on an Intel Pentium IV PC with a 1.6 GHz processor and 512 MB memory. All the interaction energies and different calculations were automatically calculated. The biological activity values IC_50_ were converted to negative logarithmic (P IC_50_) and used as the dependant variable for the QSAR analysis. Thirteen different molecular descriptors (independent variables) were selected and calculated for the submitted structures aiming cover a wide range of different electronic, hydrophobic and topological characters. The correlation matrix was calculated to avoid multicolinearity between the calculated descriptors. The correlation matrix indicated that some of the descriptors used are highly correlated which suggests avoiding the combinations between such intercorrelated descriptors (|r| ≥ 0.80, where r is the simple linear coefficient). QSAR model was then constructed after ensuring reasonable correlation of antitumor activity with individual descriptor and minimum inter-correlation among the descriptors used in derived model.

#### 3.4.2. Statistical Analysis

Stepwise linear regression analysis (SLRA) technique was used to test the best structural predictors for activity. For the current dataset of the new compounds, the QSAR model development was restricted to a maximum of three variables in accordance to the general accepted rule for the compounds: descriptors ratio to be around 5:1. The developed QSAR models are evaluated using the following statistical measures as root mean square error (RMSE) and correlation coefficient (*R^2^*) and validated by the leave-out technique (LOO technique), where each object of the data set is taken away, one at a time. In this case, given n objects, n reduced models are developed. The predicted activities (*p* IC_50_) for the tested compounds calculated using multi-linear regression ($Pred) technique.

## 4. Conclusions

Various substituted imidazo[1,5-*a*]quinazoline derivatives were synthesized. The structure of the newly synthesized compounds was elucidated by elemental analyses and spectral data. All compounds were tested, *in vitro*, for their antitumor activity. Compound **5a** is the most active against the human mammary carcinoma cell line (MCF7). Physico-chemical parameters revealed that most of the compounds obeyed the “rule of five” properties with good absorption percentages. 2D-QSAR studies helpto explore some expectedly potent compounds.
